# 铁死亡相关通路在急性髓系白血病中的研究进展

**DOI:** 10.3760/cma.j.issn.0253-2727.2023.06.019

**Published:** 2023-06

**Authors:** 颜婷 李, 艺尹 陈, 杨 徐, 德沛 吴

**Affiliations:** 苏州大学附属第一医院，国家血液系统疾病临床医学研究中心，江苏省血液研究所，国家卫生健康委员会血栓与止血重点实验室，苏州大学造血干细胞移植研究所，苏州 215006 The First Affiliated Hospital of Soochow University; National Clinical Research Center for Hematologic Diseases; Jiangsu Institute of Hematology; Key Laboratory of Thrombosis and Hemostasis Under Ministry of Health; Institute of Blood and Marrow Transplantation, Soochow University, Suzhou 215006, China

铁死亡作为一种区别于细胞凋亡、自噬、坏死等的新型调节性细胞死亡方式，与癌症存在天然联系。研究显示，诱导铁死亡在多种恶性肿瘤中展现出抗癌潜力，对传统治疗有耐药性的肿瘤细胞可能因氧化还原系统高代谢而对这种死亡方式更加敏感[1]。有证据表明，急性髓系白血病（AML）细胞系对铁死亡诱导剂erastin相较其他肿瘤细胞类型更敏感[2]–[3]。因此，靶向铁死亡有望成为治疗AML的新途径，甚至可能为难治/复发AML患者提供新的治疗机会。本文对铁死亡在AML中的研究进展进行综述。

一、铁死亡调节过程

细胞发生铁死亡的过程可简要概括为：细胞内铁死亡执行系统及防御系统的失衡，最终导致脂质过氧化物的致命性积累而诱发细胞死亡[4]。发生铁死亡时，细胞内铁积累、活性氧（Reactive oxygen species, ROS）及过氧化脂质增多，同时可伴有抗氧化系统谷胱甘肽（Glutathione，GSH）和谷胱甘肽过氧化物酶4（Glutathione peroxidase 4，GPX4）等的表达量降低[1]。

1. 铁死亡执行系统：脂质过氧化是铁死亡的关键过程。掺入磷脂双分子层的多不饱和脂肪酸（PUFA-PL）是参与铁死亡的主要脂质成分，而单不饱和脂肪酸（MUFA）则可通过竞争性抑制PUFA来抑制铁死亡发生[5]。PUFA合成过程中的酶以及AMPK通路等均可通过调节脂质合成间接调控铁死亡[5]–[6]。

铁代谢及线粒体代谢则参与PUFA-PL的氧化。线粒体代谢是生产脂质氧化所需ROS的主要途径[7]。而铁不仅通过催化芬顿反应介导ROS生成，同时还是脂质过氧化过程中多种代谢酶的重要辅助因子[7]。通过调节细胞内不稳定铁池（Labile iron pools，LIP）来增加ROS水平是铁死亡干预途径之一。

2. 铁死亡防御系统：在铁死亡中最具特征的抗氧化系统是System Xc^−^-GSH-GPX4轴。System Xc^−^是一种谷氨酸/胱氨酸转运蛋白，按1∶1的比例将胱氨酸交换至胞内。胱氨酸进一步催化生成的GSH可辅助铁死亡的中枢抑制因子GPX4削弱脂质过氧化物毒性，维持膜脂质双分子层稳态，抑制铁死亡[1]。

辅酶Q10（Coenzyme Q10，CoQ10）以及四氢生物蝶呤（Tetrahydrobiopteromine，BH4）可通过捕获ROS减少脂质过氧化。与之相关的FSP1-CoQ10轴、DHODH-CoQ10轴和GCH1-BH4轴独立于GPX4负向调节铁死亡[4]。另外，抗氧化过程中的关键转录因子Nrf2也可直接或间接参与调控多种铁死亡抑制通路，提高细胞抗氧化能力，限制ROS的产生，抑制铁死亡[8]。

二、AML中铁死亡相关调控机制

铁的增加可促进ROS的生成，而AML病程中铁过载并不少见。AML细胞铁的吸收增加，铁的流出减少，并且转铁蛋白、转铁蛋白受体、铁代谢相关蛋白也可见不同程度的异常[9]–[10]。治疗过程中为改善贫血而反复输血是铁过载的主要原因，病态造血（尤其骨髓增生异常综合征转化AML）、化疗和造血干细胞移植造成的红细胞溶解破坏和生成受抑等也会进一步加重铁过载[10]。此外，白血病细胞（尤其是白血病干细胞，LSC）主要依赖线粒体氧化磷酸化的代谢特点[11]，一些AML相关的分子突变如FLT3、NRAS/KRAS和IDH1/2等也会影响ROS的产生[12]。这种高ROS状态使得AML细胞对铁死亡具有潜在靶向脆弱性。因此通过增加细胞LIP水平来诱导AML细胞铁死亡似乎是较为理想的方式。

（一）铁稳态调节相关途径

1. 自噬-铁蛋白-LIP途径：多项研究提示自噬参与铁死亡的发生，铁蛋白是自噬介导铁死亡的主要底物。自噬体降解铁蛋白，使其释放出所结合的铁，增加LIP及ROS水平，诱导铁死亡的发生。

双氢青蒿素（Dihydroartemisinin, DHA）是青蒿素的衍生物，具有抗疟、免疫调节作用及抗肿瘤活性，并可通过调节AMPK-mTOR-p70S6k途径激活自噬，介导AML细胞铁死亡[13]。一种植物提取物香蒲新苷（Typhaneoside）也可通过AMPK-mTOR途径在AML中诱导自噬介导的铁死亡，抑制自噬后可恢复细胞活力[14]。

由核受体共激活因子4（Nuclear receptor coativator 4，NCOA4）介导的铁蛋白降解又被称为铁自噬。新型全反式维甲酸衍生物ATPR可通过调节Nrf2激活ROS-自噬-溶酶体信号传导，由NCOA4介导铁自噬诱导铁死亡，而抑制这种铁死亡有助于ATPR诱导的AML分化[15]。由于铁及ROS水平均可影响AML细胞增殖和分化，该研究也提示靶向铁死亡可能帮助调节AML疾病进展。

2. RAS-JNK/p38-TfR1-LIP途径：MAPK包括ERK、JNK、p38等亚族，此前的研究表明，携带RAS突变的纤维肉瘤及肺癌细胞可发生RAS-ERK信号通路介导的铁死亡，但在HL-60细胞中，erastin通过RAS-JNK/p38途径增加转铁蛋白受体1（Transferrin receptor 1，TfR1）的表达，继而增加LIP水平，介导铁死亡[3],[16]。在该通路中，高迁移率族蛋白1（High mobility group protein 1, HMGB1）作为调控因子起关键作用，可能成为AML治疗干预的潜在药物靶点[16]。

（二）GPX4相关途径

System Xc^−^-GSH-GPX4通路是铁死亡的经典调节途径。erastin和柳氮磺胺吡啶可通过抑制System Xc^−^来诱导AML细胞铁死亡，同时导致细胞内GSH水平降低、GPX4活性受抑，因此推测它们可能通过System Xc^−^-GSH-GPX4通路诱导AML细胞铁死亡，但还需进一步实验佐证[17]。p53也可通过抑制SLC7A11（System Xc^−^的组成蛋白）的转录参与AML铁死亡调节[18]。

GPX4是铁死亡中枢调节因子，铁死亡抑制剂RSL3靶向抑制GPX4来诱导人髓系白血病细胞株MOLM-13铁死亡，伊达比星及阿糖胞苷耐药的MOLM-13细胞株对GPX4的抑制更为敏感[19]。最新研究发现在AML细胞中可通过非经典途径调节GPX4来诱导铁死亡。重组人促血小板生成素（rhTPO）通过结合白血病细胞中的E1A结合蛋白P300（EP300），抑制EP300增强GPX4基因转录的作用，诱导铁死亡。结合之前研究，EP300与磷酸化CREB蛋白特异性结合来介导cAMP基因调控，增强GPX4基因转录以抑制铁死亡。推测rhTPO通过竞争性结合EP300来抑制其与CREB的相互作用，从而介导AML细胞铁死亡[20]。

（三）PI3K-AKT途径

对在AML中差异表达的铁死亡相关基因（Ferroptosis-related Genes, FRG）进行KEGG通路分析显示PI3K-AKT信号通路显著富集，该通路主要调节代谢、增殖、细胞存活和血管生成[21]–[22]。既往研究显示PI3K-AKT-mTOR信号通路的持续激活上调SREBP1/SCD1，促进MUFA合成，使得癌细胞抵抗铁死亡，联合使用PI3K-AKT-mTOR通路拮抗剂及铁死亡诱导剂可显著清除乳腺癌和前列腺癌小鼠体内肿瘤[23]。PI3K-AKT-mTOR通路介导FLT3突变AML中白血病细胞的存活，但单独应用PI3K-AKT-mTOR抑制剂临床效果不佳[24]，提示将该通路作为铁死亡干预靶点或许有利于改善在AML（尤其是FLT3突变的AML）中的疗效。

三、AML中铁死亡的预后意义

AML耐药及复发是影响预后的重要因素，靶向铁死亡展现出的抗白血病作用无疑为难治/复发型AML患者带来新希望。此外，FRG也有望作为AML诊断、治疗及预后的新生物标志物。

基因突变是AML独立预后因素，分子生物学技术及流式细胞术的发展可辅助探索更详尽的AML分子背景，以期实现精准预后。近年来，FRG在AML中的预后意义和功能得到越来越多的关注，部分研究通过筛选与AML患者总生存（OS）期相关的FRG来构建预后模型（表1）。通过这些模型划分出的高风险组患者相较低风险组患者的OS率明显降低。随后的功能分析显示，高、低风险组在免疫状态及药物敏感性等方面也存在差异。此外，尽管不同研究筛选出的FRG存在差异，但这些FRG在功能上多涉及抗氧化（如AIFM2、SLC7A11、CD44）、脂质代谢（如ACSF2、GPX4）、铁代谢（如CISD1）、癌症代谢（SOCS1、DPP4）等方面。

**表1:** 预后模型中铁死亡相关基因（FRG）

文献来源	年份	FRG来源	FRG
Zheng等[17]	2022	FerrDb	ACSF2、AIFM2、EIF2AK4、GPX4、MYB、SLC7A11、SOCS1
Song等[21]	2022	文献检索	ACSF2、AIFM2、CHAC1、CISD1、DPP4、GPX4、PGD、SQLE
Zhu等[25]	2022	文献检索	ACSF2、AIFM2、CD44、CHAC1、CISD1、DPP4、G6PD、NCOA4、SAT1、SLC7A11
Huang等[26]	2022	GeneCards、BioGPS	ACSL5、ACSL6、AIFM2、CD44、CISD1、FH、G3BP1、GPX4、HSPB1、LPCAT3、SESN2、SOCS1
Shao等[27]	2021	FerrDb	ACSL3、ARNTL、CXCL2、DDIT4、ENPP2、HSD17B11、HRAS、PGD、PHKG2、PSAT1、SLC38A1、STEAP3
Cui等[22]	2022	FerrDb	ATG3、FAM106A、KLHL9、LCMT2、LRRC40、LZTR1、NCR2、PAFAH2、PCMTD2、PLA2G5、SCARB1、TK1、ZNF576
Wang等[28]	2022	FerrDb、文献检索	ASTN1、CCL23、DLL3、EFNB3、FAM155B、FOXL1、HMX2、HRASLS、LGALS1、LHX6、MXRA5、PCDHB12、PRINS、TMEM56、TWIST1、ZFPM2、ZNF560、ZSCAN4

虽然仅通过FRG构建的预后模型较为片面，但这些模型中所涉及的关键风险基因可作为潜在预后生物标志物，帮助深入理解AML分子基础，改善危险分层，构建更精准AML预后系统。相关的免疫及药敏数据也可帮助筛选目标人群实现精准治疗，优化临床决策[17],[2]–[22],[25]–[28]。

四、治疗

铁死亡过程中的铁代谢、线粒体代谢及抗氧化系统调节等均是重要靶点。目前已知的铁死亡诱导剂（Ferroptosis inducers，FIN）大致分为四种类型：Ⅰ类FIN靶向抑制System Xc-，Ⅱ类和Ⅲ类FIN直接或间接抑制GPX4，Ⅳ类FIN通过增加LIP水平介导铁死亡[29]。很多可通过介导铁死亡来抗白血病的药物多也是直接或间接通过上述途径起效的。但要作为单一药物使用，还需进一步探索AML对特定FIN的靶向脆弱性。还要注意的是，部分经典FIN如erastin因代谢不稳定及溶解度有限，并不适用于临床[2]。

另一方面，铁死亡被认为是多种肿瘤治疗方式引起的细胞死亡方式，通过调节铁死亡可逆转肿瘤对化疗、靶向治疗和免疫治疗等治疗手段的耐药性并帮助增强疗效[30]。但事实上，现阶段铁死亡相关研究向临床转化仍任重道远，相比之下探讨当下临床用药的铁死亡调节相关抗白血病作用，以及AML中铁死亡干预与传统肿瘤治疗手段的联合可能更具有临床转化意义。

1. 调节铁死亡联合化疗：虽然erastin并不适用于临床，但实验室数据提示低浓度erastin诱导的铁死亡可增强AML细胞对化疗药物阿糖胞苷和阿霉素的敏感性[3]。柳氮磺胺吡啶可通过抑制System Xc-活性诱发AML铁死亡而表现出抗白血病作用，并且显著增强柔红霉素和阿糖胞苷组合的疗效，这些结果在45个NPM1突变AML样本及患者来源的异种移植模型中也得到验证[31]。柳氮磺胺吡啶已被批准用于炎症性肠病和类风湿性关节炎的治疗，上述结果提示未来将其与化疗结合应用于AML治疗的可行性。

化疗与铁死亡诱导相结合对LSC也表现出明显抗癌效果。LSC在AML耐药和肿瘤复发等方面起着至关重要的作用。通过线粒体自噬、抑制抗氧化酶等途径维持低代谢及低ROS水平是LSC耐药机制之一[11]。研究发现氧化铁纳米颗粒IONP与阿糖胞苷联合治疗可明显提高LSC内ROS水平，有效降低LSC移植小鼠的肿瘤负荷并延长小鼠存活期[32]。靶向铁死亡联合化疗对AML细胞及LSC的作用在完全根除肿瘤方面显示出一定潜力。

2. 调节铁死亡联合免疫治疗：程序性死亡配体1（PD-L1）在包括AML在内的多种肿瘤中上调，并通过结合T细胞上的程序性死亡受体1（PD-1）来抑制细胞毒性T细胞活性。通过阻断PD-1/PD-L1的免疫检查点阻断（Immune checkpoint blockade，ICB）疗法在癌症治疗研究方面取得了很大进展，但耐药问题使得实际疗效不尽如人意[33]。研究显示调节铁死亡有助于克服PD-1/PD-L1治疗耐药性[33]，而免疫疗法在激活CD8^+^T细胞的同时可通过下调SLC7A11的表达来介导铁死亡[34]。研究者设计了一种基于甘草次酸的纳米平台（GCMNP），甘草次酸作用于线粒体呼吸链产生ROS来诱导铁死亡，GCMNP搭载一种氧化铁纳米颗粒ferumoxytol后加强了铁死亡毒性作用，而GCMNP+ferumoxytol+PD-L1抗体的组合明显改善了T细胞在白血病细胞中的免疫反应。该研究提供了一种结合铁死亡及ICB诱导的抗白血病肿瘤免疫新策略[35]。

3. 调节铁死亡联合放疗：放疗也与铁死亡之间存在紧密联系。研究显示靶向铁死亡可使放疗增敏，改善放射抵抗[30]。虽然相关研究仍有待进一步深入，但通过这种联合来增强疗效并减少放射剂量和不良反应的潜能无疑是值得期待的。另外，除了造血干细胞移植前的常规放疗，放疗多用于难治/复发或髓外浸润的白血病患者，这些患者因一般情况较差而更可能从这种联合中获益。

五、总结

铁死亡作为一种新型程序性细胞死亡方式在包括AML在内的多种肿瘤治疗中显示出巨大应用前景。目前研究提示通过自噬相关、RAS-MAPK等通路干预铁代谢，GPX4相关通路调节抗氧化系统等途径可诱导白血病细胞铁死亡，甚至在耐药细胞株及LSC中也显示出一定疗效，为AML（尤其是难治/复发型）的治疗提供新思路。但目前AML对单药诱导铁死亡的脆弱性仍有待进一步探索，铁死亡诱导与化疗、免疫治疗等传统抗肿瘤治疗方式的联合可能更具有临床转化意义。期待未来对相关机制及临床转化的深入探讨能为更多患者带来新希望。
